# Recent advances in the roles of exosomal microRNAs in neuroblastoma

**DOI:** 10.3389/fonc.2022.1091847

**Published:** 2023-01-10

**Authors:** Swapnil Parashram Bhavsar

**Affiliations:** Pediatric Research Group, Department of Clinical Medicine, Faculty of Health Sciences, UiT - The Arctic University of Norway, Tromsø, Norway

**Keywords:** exosomal miRNAs, metastasis, neuroblastoma, biofluids, tissues and conditioned medium

## Abstract

Exosomal miRNAs (exo-miRs), universally found in biofluids, tissues, and/or conditioned medium of the cell cultures play a significant role in cell - cell communication, thus driving cancer progression and metastasis. Very few studies have explored the role of exo-miRs in the progression of children’s cancer - neuroblastoma. In this mini review, I briefly summarize the existing literature on the role of exo-miRs in the pathogenesis of neuroblastoma.

## Introduction

### Neuroblastoma

Neuroblastoma is the most common extra cranial solid tumor in children derived from the primitive cells of the sympathetic nervous system (1). It accounts for nearly 15% of pediatric oncology deaths (2). High-risk neuroblastoma is often manifested with *MYCN* amplifications, which is one of the most reliable prognostic factors. Patients with *MYCN* amplifications have poor prognosis and high mortality (3). Treatment of neuroblastoma mainly involves surgery and chemotherapy. However, despite rigorous treatment approaches, there are high chances of tumor relapse and treatment failure in high risk neuroblastoma patients. Acquired drug resistance is one of the major obstacles in the treatment failure (4). Therefore, new approaches are warranted to combat this lethal disease.

### MicroRNAs

MiRNAs are a large family of small, endogenous, non-coding RNAs, which target messenger RNAs (mRNAs) regulating the expression of genes, resulting in the development and progression of the physiological processes (5). Aberrant expression of miRNAs is shown associated with variety of diseases (6). Due to advent of high-throughput sequencing, new miRNAs are being discovered (7) and are shown to play significant role in cancer development and progression (5). Ever since the discovery of miRNAs in 1993 (8, 9), thousands of miRNAs have been characterized. The miRBASE version enlist more than 11000 miRNAs (7). Growing evidence have shown the potential of miRNAs as prognostic and diagnostic markers of human malignancies (10). Recently, miRNAs secreted in the extracellular vesicles are shown to have important roles in cancer metastasis, drug resistance (11, 12) and pre-metastatic niche formation (13).

### Exosomes: Biogenesis and functions

Exosomes are a subclass of extracellular vesicles derived from the cellular origin. They are membrane-enclosed nanoscale particles of about 40-160nM in size. The exosome cargo mainly constitutes - lipids, proteins, and nucleic acids (DNA and RNA) (14). Owing to its lipid bilayer membrane enclosed structure, exosome contents are protected from degradation in the extracellular space. This helps in very efficient cell-cell communication (15). ExoCarta, a manually curated web-based compendium of exosomal RNAs, proteins and lipids contain 3408 mRNAs, 9769 proteins and 1116 lipids based on 286 studies on exosomes (16).

The process of exosome biogenesis begins with the inward budding of the plasma membrane, to form a structure called as early endosomes. Early endosomes subsequently mature to form late endosomes. And during this maturation process, RNA, protein, and lipid cargo is incorporated into the intraluminal vesicles (ILVs). The late endosomal structures contain dozens of ILVs and are therefore called multivesicular bodies (MVBs). These MVBs eventually fuse with the plasma membrane, wherein the ILVs are released as exosomes in the extracellular space *via* a process called exocytosis. Variety of cellular components are involved in the biogenesis and secretion of exosomes into the extracellular space. The main role is played by endosomal sorting complex required for transports (ESCRTs) machinery. The ESCRTs (0, I, II, and III) form complexes with associated proteins (ALIX, VTA1, VPS4 and TSG101) enabling vesicle budding and cargo sorting in MVBs (15, 17–19).

**Figure 1:**
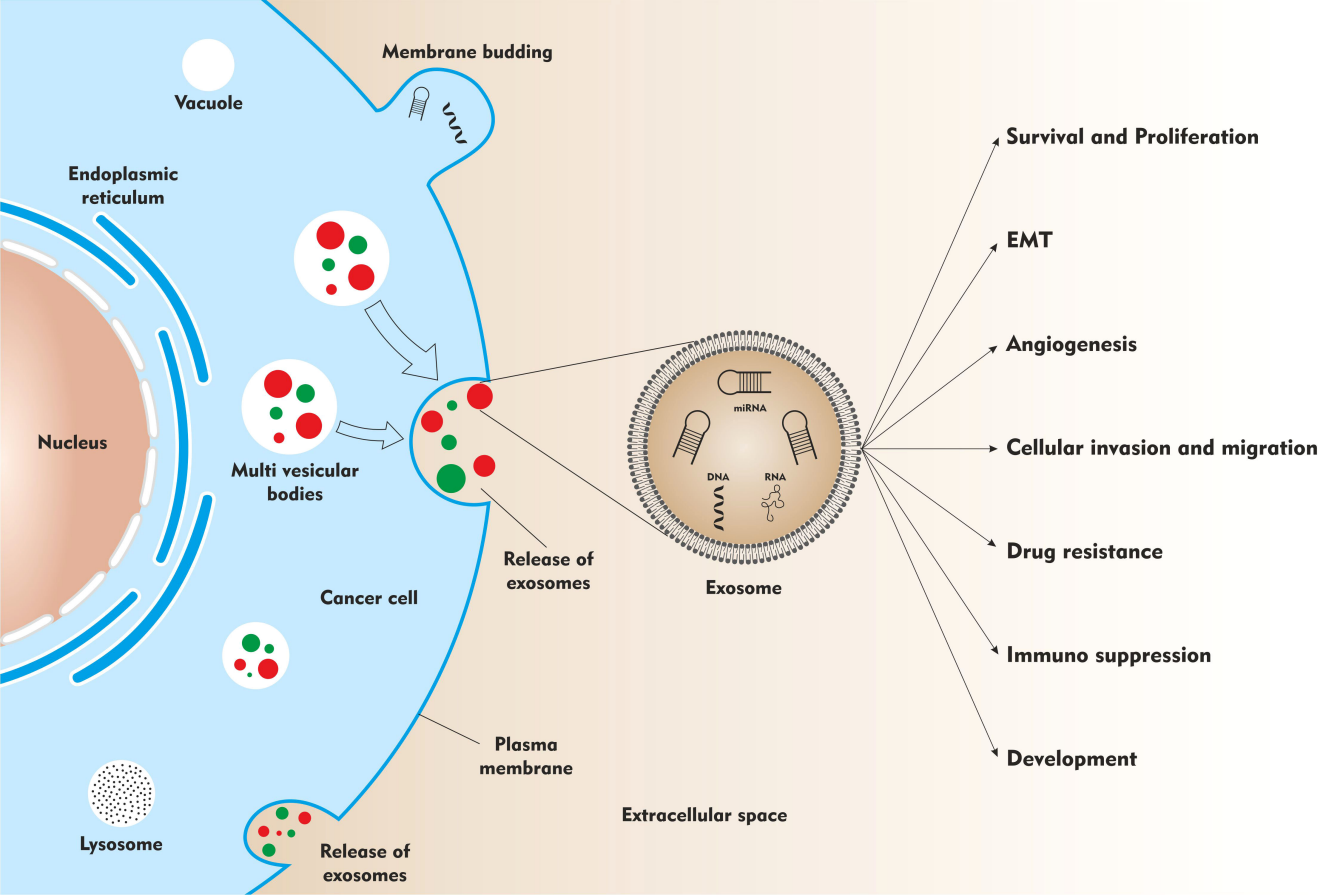
The role of exosomes in neuroblastoma development and progression.

Exosomes play a significant role in the regulation of the pathophysiological processes (**Figure 1**). They mediate intercellular communication *via* the transfer of genetic information from donor cells to the recipient cells. The exosome cargo may contain different types of transcription factors, oncogenes, messenger RNAs, miRNAs, and infectious particles. Due to their natural property of genetic information transfer, they exert pleiotropic effects on target cells resulting in exchange of information between cells. Exosomes are also shown to mediate epigenetic changes in the target cells (20–22)

### Exosomal miRNAs in neuroblastoma

Here, I discuss the roles of exo-miRs in neuroblastoma, with the most relevant scientific publications available on the PubMed website. Very few studies have discussed the role of exo-miRs in neuroblastoma. I searched the key words, “exosomes”, “miRNAs”, and “neuroblastoma”, in the PubMed search option in August 2022, which produced 24 results (https://pubmed.ncbi.nlm.nih.gov/). I then selected seven relevant publications for further analyses and discussion.

In a first, our laboratory has shown that, MYCN-amplified neuroblastoma cells secreted exosome-like extracellular vesicles whose cargo contain oncogenic miRNAs. Ready-to-use qPCR panels (LNA-qPCR arrays measuring 742 miRNAs) employed to profile exo-miRs from the two MYCN amplified neuroblastoma cell lines - SK-N-BE(2)-C and Kelly. We compared the top 25 highly expressed exo-miRs and 11 (miR-16-5p, miR-125b-5p, miR-21-5p, miR-23a-3p, miR-24-3p, miR-25-3p, miR-27b-3p, miR-218-5p, miR-320a, miR-320b and miR-92a-3p) of these were common to both cell lines and included several known oncogenic miRNAs. We also demonstrated the internalization of exosomes into the recipient cells. Furthermore, a functional enrichment analysis using predicted mRNA target genes from the 25 highly expressed exo-miRs from MYCN-amplified neuroblastoma cell lines revealed pathways related to cell growth, development, survival, and death. The top score was obtained for AHR (Aryl Hydrocarbon Receptor) signaling pathway, which is inversely correlated to MYCN expression in neuroblastoma tissue. The AHR has been shown to be upstream regulator of MYCN (23).

A study by Challagundla and colleagues, demonstrated a significant role of exo-miRs - miR-21 and miR-155, in the cross talk between neuroblastoma cells and human monocytes (in the tumor microenvironment) affecting drug resistance. qRT-PCR screening of exo-miRs secreted by five different neuroblastoma cell lines - SKNBE(2), CHLA-255, IMR-32, LA-N-1 and KNCR, led to identification of miR-21, which was the highly secreted miRNA in all the cell lines. Co-culture experiments demonstrated exosomal miR-21 transfer from neuroblastoma cells to human monocytes, which led to TLR8 and NF-kB dependent upregulation of miR-155. In turn, exosomal miR-155 from human monocytes re- transferred to neuroblastoma cells. MiR-155 directly targets TERF1 and affect telomerase activity and telomerase length in neuroblastoma. Interestingly, telomere length and telomerase activity are correlated with drug resistance and poor outcome in various cancers (24). MiR-155 and TERF1 expression levels were also assessed in 20 primary neuroblastoma tissues. Higher expression of miR-155 and lower levels of TERF1 was observed in neuroblastoma tissues with higher TAM infiltration (25).

Neviani and colleagues showed that natural Killer (NK) cells secrete cytotoxic exosomes, which carry tumor suppressor miR-186. MiR-186 was down-regulated in high-risk neuroblastoma. Further analysis of miR-186 revealed targets including MYCN, AURKA and several TGFB-pathway members like TGFBR1, TGFBR2, SMAD2 and SMAD3 important for survival and immune escape mechanisms in neuroblastoma. Evaluation of miR-186-5p in publicly available datasets demonstrated lower expression of miR-186 in high-risk and MYCN amplified neuroblastoma and correlated with expression of NK activation markers, NKG2D and DNAM-1. Functional assays with forced expression of miR-186 in neuroblastoma cell lines and NK cells, inhibited survival, and migration both *in vitro* and *in vivo* and prevented TGFB1 dependent inhibition of NK cytotoxicity. Interestingly, when the abundance level of miR-186 was altered in NK-derived exosomes; the exosomal cytotoxic potential also altered. Furthermore, to test if the *in vivo* delivery of miR-186 could serve as a possible anti-cancer strategy, an orthotopic mouse model of neuroblastoma was used for targeted delivery of miR-186 to the tumor site. This led to significant reduction of tumor burden and improved survival (26).

The study by Ma and colleagues, proposed plasma exosomal hsa-miR-199a-3p as a non-invasive diagnostic and prognostic biomarker for neuroblastoma. MiRNA profiling of exosomes by next-generation sequencing (NGS) performed on plasma of 17 neuroblastoma (NB), 6 intermixed ganglioneuroblastoma (GMBi) and 7 healthy controls (HCs). Differential Gene Expression (DEG) analysis of sequencing data revealed 3,779 differentially expressed exo-miRs, among which 3,248 miRNAs were up-regulated and 531 miRNAs were down-regulated in the NB/GNBi patients. The plasma exosomal hsa-miR-199a-3p was significantly upregulated in NB/GNBi patients as compared to healthy controls and correlated with high-risk neuroblastoma. They also showed that exosomal hsa-miR-199a-3p could target NEDD4 in neuroblastoma cells and consequently promote tumor proliferation and migration (27).

In another study, Morini and colleagues tested the potential of exo-miRs in blood to predict highrisk neuroblastoma (HR-NB) patient’s response to induction chemotherapy. Exosomes were isolated and characterized from 52 HR-NB patients before and after induction chemotherapy. Interestingly, they observed significant reduction of NB-derived exosomes after the chemotherapy treatment. Next, to investigate the modulation of exo-miRs upon chemotherapy, the exosomal miRNA expression profiling was done by RTqPCR, which led to differential expression of exo-miRs before and after induction chemotherapy. Analysis of the differentially expressed miRNAs led to identification of a three exo-miRs signature (miR-342-3p, miR-29c and let-7b), which could discriminate between poor and good responders. Furthermore, pathway analysis of the exo-miRs signature demonstrated their association with pathways playing important role in tumor progression and chemoresistance (28).

Colletti and colleagues proposed that exosomal miR-375 is involved in neuroblastoma infiltration of bone marrow (BM). They demonstrated that bone marrow mesenchymal stromal cells (BM-MSCs) isolated from neuroblastoma patients with bone marrow infiltration or metastasis has greater osteogenic potential then BM-MSCs isolated from neuroblastoma patients without bone marrow infiltration or metastasis and from healthy controls (HCs). Next, they showed BM-metastasis derived neuroblastoma cell lines (SK-N-SH, SH-5Y-SY, SKNBE(2)-C, LAN-1 and IGR-N91) had increased levels of exosomal miR-375, which promotes osteogenic differentiation in MSCs, favoring survival and proliferation of metastatic cells. Moreover, the clinical data derived from 60 neuroblastoma patients revealed positive correlation between increased expression of miR-375 and BM infiltration. This study thus proposes that, evaluation of miR-375 in blood and BM would be useful for diagnosis in neuroblastoma patients (29).

In yet another study, Chen carried out experiments to check if exosomes could transfer oncomiRs and the cancerous phenotype to other cells. Analysis of three independent published studies demonstrated that miR-17-92 cluster expression in MYCN amplified neuroblastoma tissues was considerably higher than non-MYCN amplified neuroblastoma. Likewise, the expression level of miR-17-92 cluster and in particular miR-17-5p, in MYCN amplified SKNBE(2) was considerably higher than non-MYCN amplified SH-SY-5Y. Thus, the exosomes derived from SKNBE(2) cells (with higher miR-17-5p expression) were co-cultured with SH-SY-5Y cells, which resulted in up-regulation of miR-17-5p in SH-SY-5Y cells. In SH-SY-5Y cells, miR-17-5p targets PTEN and activates PI3K/AKT signaling which enhances the proliferative and migratory abilities of SH-SY-5Y cells (30).

## Discussion

In this mini review, I discuss the role of exo-miRs in the context of neuroblastoma.

As analyzed in the earlier section, oncogenic miRNAs (23, 27, 29, 30) and tumor suppressor miRNAs (26) are secreted and transported as exosomal cargo in cell culture conditioned media or in blood to mediate its effect locally or at distant sites. These exo-miRs are shown to functionally affect - tumor survival, proliferation, migration, and drug resistance (**Figure 1**).

Tumor microenvironment (TME) plays a very important role in tumor progression and drug resistance (31). For example, Challagundla and colleagues explored unique mechanism of miRNA transfer from cancer cells to non-cancerous cells in the TME. Exo-miRs were transferred between neuroblastoma cells and human monocytes, which led to drug resistance in neuroblastoma (25). In another study, innate cytotoxic immune cells like natural killer-NK cells (present in the TME) also secrete exosomes, whose miRNA content affected tumor burden in neuroblastoma (26).

Pre-metastatic niche (PMN) is a ground well prepared for homing of the tumor cells (32). Colletti and colleagues demonstrated the role of exosomal miR-375 in defining the bone marrow PMN formation for bone marrow metastasis (29).

In these studies analyzed, molecular biology techniques like real time-quantitative polymerase chain reaction (RTqPCR) and next generation sequencing (NGS) (27) employed to detect and quantify miRNAs present in exosomes - recovered from the conditioned media of neuroblastoma cell lines, blood plasma and/or immune cells (see **Table 1**). Functional *in vitro* and *in vivo* assays performed by enforced expression of miRNA mimics or antagomirs to test and validate their predicted targets and phenotypic effects. Moreover, publicly available neuroblastoma datasets assessed to evaluate miRNA expression patterns and correlations with prognostic factors.

**Table 1:** The articles investigating the role of exosomal miRNAs (exo-miRs) in neuroblastoma.

Source of exosomes	Exosome isolationtechnique	Exosomal miRNA expression profiling	Exosomal miRNAs detected in neuroblastoma	Functional effects	Reference
Conditioned media from SK-N-BE(2)-C and Kelly	Ultra- centrifugation	RT-qPCR	miR-16-5p, miR-125b-5p, miR-21-5p, miR-23a-3p, miR-24-3p, miR-25-3p, miR-27b-3p, miR-218-5p, miR-320a, miR-320b and miR-92a-3p	Proliferation	23
Conditioned media from SK-N-BE(2), CHLA-255, IMR-32, LA-N-1, KNCR	ExoQuick™ solution	RT-qPCR	miR-21 and miR-155	Proliferation and Drug resistance	25
Conditioned media from NK cells	Size exclusion chromatography	RT-qPCR	miR-186	Proliferation and Migration	26
Plasma	exoRNeasy Serum/Plasma Midi kit	NGS	miR-199a-3p	Proliferation and Migration	27
Plasma	exoRNeasy Serum/Plasma Midi kit	RT-qPCR	miR-342-3p, miR-29c, let-7b	Tumor progression and drug resistance	28
Conditioned media from SK-N-SH, SH-5Y-SY, SKNBE(2)-C, LAN-1 and IGR-N91	Sequential Centrifugation	RT-qPCR	miR-375	Proliferation	29
Conditioned media from SK-N-BE(2)	Ultra- centrifugation	RT-qPCR	miR-17-5p	Proliferation and Migration	30

Exosome isolation techniques like sequential centrifugation and commercially available kits used in these studies (see **Table 1**). The methods of isolation and profiling differ, which may result in different expression profiles from the same sample. Therefore, consistent, and standard use of a method would increase intramethod reproducibility. Standardization of methods for isolation, quantification and analysis could ease interlaboratory comparison of data. The potential candidate biomarkers resulted from various profiling methods should be functionally validated in multiple independent study populations.

Multiple studies have demonstrated that single miRNA can target many genes (33) and many miRNAs are present in the exosomes (34), so the effect of single miRNA cannot be generalized. Moreover, interactions of exo-miRs with each other should be considered. Studies have shown either one or two targets affected by exo-miRs (35). However, other potential targets should also be evaluated critically.

Most of the experiments performed to delineate the role of exo-miRs are conducted using either one or two neuroblastoma cell lines. However, this could be a limitation considering the heterogenetic nature of neuroblastoma (36). Many different neuroblastoma cell lines along with patient derived xenograft (PDX) tissue samples and experiments using mouse models should be carried out to validate the results.

As observed in these studies, few cross-sectional studies and only one longitudinal study was performed in relation to exo-miRs in neuroblastoma. More longitudinal studies are required considering different aspects of neuroblastoma progression. Additionally, most of the literature is focused on conducting *in vitro* experiments on neuroblastoma cells rather than using clinical specimens from neuroblastoma patients. Furthermore, sample size is another limitation in several studies. Hence, studies involving more patients from different populations are required to validate the findings.

### Therapeutic utility of exosomal miRNAs

Exosomes are found in all types of body fluids and the fact that, exosome can be obtain non-invasively, makes them very interesting candidates as biomarkers of tumor diagnosis and prognosis (37, 38). They play vital role in reprogramming of the target cells thus affecting tumor growth and progression. Tumor and stromal cells interact in the tumor microenvironment (39). Therefore, not only tumor cells derived exo-miRs, but also stromal cells derived exo-miRs could play important role in clinical diagnosis and treatment.

## Conclusions and future perspectives

Extracellular vesicles like exosomes have attracted much attention as potential vehicles for drug delivery and therapy response (40). Exosome primarily function in cell-to-cell communication (41). They play major role in bidirectional transfer of molecules between tumor cells and the microenvironment. It is well established that exosomes are responsible for alteration of normal environment to pro-tumorigenic environment (42). Thus, targeting exosomes or inhibiting the ability of cells to receive signals from exosomes could interrupt cancer progression and metastasis. Increasing number of studies are focusing on the tumor-derived exo-miRNA mediated intercellular cross talk and remodeling of the tumor microenvironment (43). Comprehensive understanding of the underlying mechanisms is necessitated to combat this deadly disease. There are yet other challenges, which needs to be addressed. Some studies have shown selective encapsulation of specific miRNAs in exosomes (44). In addition, the components of exosomes like RNA, protein, lipids can interact or co-operate among each other, which can lead to favorable microenvironment for cancer development and metastatic spread. These mechanisms of selective encapsulation and interactions of exosomal components are not fully understood and needs further investigation.

## Author contributions

The author confirms being the sole contributor of this work and has approved it for publication.
